# Brain Metastasis from Unknown Primary Tumour: Moving from Old Retrospective Studies to Clinical Trials on Targeted Agents

**DOI:** 10.3390/cancers12113350

**Published:** 2020-11-12

**Authors:** Roberta Balestrino, Roberta Rudà, Riccardo Soffietti

**Affiliations:** 1 Department of Neuroscience, University of Turin, Via Cherasco 15, 10121 Turin, Italy; 2 Department of Neurology, Castelfranco Veneto/Treviso Hospital, Via dei Carpani, 16/Z, 31033 Castelfranco Veneto, Italy; rudarob@hotmail.com; 3 Department of Neuro-Oncology, University of Turin, Via Cherasco 15, 10121 Turin, Italy; riccardo.soffietti@unito.it

**Keywords:** brain metastasis, cancer of unknown primary, target therapy, molecular markers, gene expression profiling, neuro-oncology, clinical trial

## Abstract

**Simple Summary:**

Brain metastases (BMs) are the most common intracranial tumours in adults and occur up to 3–10 times more frequently than primary brain tumours. In up to 15% of patients with BM, the primary tumour cannot be identified. These cases are known as BM of cancer of unknown primary (CUP) (BM-CUP). The understanding of BM-CUP, despite its relative frequency and unfavourable outcome, is still incomplete and clear indications on management are missing. The aim of this review is to summarize current evidence on the diagnosis and treatment of BM-CUP.

**Abstract:**

Brain metastases (BMs) are the most common intracranial tumours in adults and occur up to 3–10 times more frequently than primary brain tumours. BMs may be the cause of the neurological presenting symptoms in patients with otherwise previously undiagnosed cancer. In up to 15% of patients with BMs, the primary tumour cannot be identified. These cases are known as BM of cancer of unknown primary (CUP) (BM-CUP). CUP has an early and aggressive metastatic spread, poor response to chemotherapy, and poor prognosis. The pathogenesis of CUP seems to be characterized by a specific underlying pro-metastatic signature. The understanding of BM-CUP, despite its relative frequency and unfavourable outcome, is still incomplete and clear indications on management are missing. Advances in diagnostic tools, molecular characterization, and target therapy have shifted the paradigm in the approach to metastasis from CUP: while earlier studies stressed the importance of finding the primary tumour and deciding on treatment based on the primary diagnosis, most recent studies focus on the importance of identifying targetable molecular markers in the metastasis itself. The aim of this review is to summarize current evidence on BM-CUP, from the diagnosis and pathogenesis to the treatment, with a focus on available studies and ongoing clinical trials.

## 1. Introduction

Brain metastases (BMs) are the most common intracranial tumours in adults and occur up to 3–10 times more frequently than primary brain tumours [1]. Population-based data reported that about 8–20% of patients with cancer are affected by symptomatic BMs, although an incidence up to 40% has been observed in autoptic series. BMs may be the cause of neurological presenting symptoms in patients with otherwise previously undiagnosed cancer. More commonly, the origin of BMs is found in the lung (20–56% of patients), breast (5–20%), and skin (melanoma) (7–16%). However, in up to 15% of patients with BMs, the primary tumour cannot be identified. These cases are known as BM of cancer of unknown primary (CUP) (BM-CUP) [1,2]. CUP is the seventh/eighth most common malignancy, accounts for 2–5% of all malignancies, and is the fourth most common cause of cancer-related deaths. CUP manifests, by definition, by metastases. It is more commonly characterised by early and aggressive metastatic spread, poor response to chemotherapy, and dismal prognosis, which has led to the hypothesis of an underlying pro-metastatic signature [3].

The understanding of BM-CUP, despite its relative frequency and unfavourable outcome, is still incomplete and clear indications on management are missing. The aim of this review is to summarize current evidence on the diagnosis and treatment of BM-CUP with particular attention paid to the most innovative approaches. 

## 2. Methods

A literature search was performed using PubMed and EMBASE by a resident neurologist (RB), supervised by a senior neurologist (RS). The search was last updated on the 12th October 2020. The search string on PubMed was: “brain metastases”[All Fields] OR “brain metastasis”[All Fields] AND ((“neoplasms”[MeSH Terms] OR neoplasm[Text Word]) OR cancer[Text Word]) AND unknown[All Fields]; in further research, the terms “clinical trial” and “target therapy” were added. The search string in EMBASE was: “brain metastasis” and “cancer of unknown primary site”; in further research, the terms “clinical trial” and “target therapy” were added. Articles were screened based on titles and abstracts, and only full-text articles in English were included. Reviews and editorials/opinions were excluded. During full-text analysis, the reference list was screened to identify additional studies not retrieved through the database search. Clinical trials were searched in the website clinicaltrial.gov using as condition/disease “unknown primary cancer”, “cancer of unknown origin”, “cancer of unknown primary”, “unknown primary neoplasm metastasis”, and “brain metastasis” and related terms; studies classified as “terminated”, “withdrawn”, and “unknown status” were excluded.

## 3. Definition and Diagnosis of CUP

CUP is defined as a histologically confirmed metastatic cancer for which clinicians are unable to identify a primary tumour source after the diagnostic workup. 

Different definitions of CUP have been used by different authors over the years, complicating the identification of a homogeneous entity in literature. BM-CUPs have been variably defined either as BM with no previously known cancer (i.e., BM as the first manifestation of a systemic cancer, with the primary site not always remaining unknown) [4,5,6,7,8,9,10,11,12,13], BM in which the primary site has not been identified within a temporal interval from onset (more often 2–3 months) [14,15,16,17,18], or as BM with no primary tumour identified after standard or extensive work-up [19,20,21,22,23,24,25,26,27,28]. In some studies, the definition of CUP is not clearly stated [29,30,31,32,33,34,35,36,37,38,39,40,41,42,43,44,45,46]. The chance of identifying a primary site through clinical and histopathological evaluation has changed over time due to improvements in imaging and surgical techniques. Thus, it is difficult to provide accurate incidence data and compare studies from different periods. Advances in technology have increased the likelihood of finding the primary tumour, probably contributing to the decrease in the incidence of CUP in general [47,48]; however, in some cases, the primary tumour remains unknown even after autopsy [49]. Overall, CUP seems to account for approximately 10–15% of BMs; however, a range between 1% and 61% is reported throughout the literature, with rates around 1% in more recent studies on large BM cohorts [8,33] as compared to 50–60% in older studies [21,28]. 

Currently, the diagnostic standard for CUP, as laid down in the European Society of Medical Oncology (ESMO) guidelines [50], includes physical examination, basic blood and biochemistry analyses, as well as computed tomography (CT) or magnetic resonance imaging (MRI) of the chest, abdomen, and pelvis, and tissue for histological characterization and immunohistochemistry (IHC); further tests should be performed based on the clinical picture and the IHC profile. In addition, in case of CUP, the European Association of Neuro-Oncology (EANO) guidelines on BM [51] recommend mammography and/or ultrasound of breast and, if negative, whole-body 18F-fluordesoxyglucose position emission tomography (FDG-PET). The primary tumour detection rate after FDG-PET in CUP ranges between 22% and 73% [52]. With regard to BM-CUP, a recent study reported a sensitivity of 85–92%; however, the comparison between FDG-PET/CT and chest/abdomen CT did not demonstrate any difference in localising the primary lesion, probably because most primary tumours were located in the lung. However, FDG-PET/CT improved the accuracy in detecting other metastases, thus decreasing the graded prognostic score (GPA) in the prediction of survival [11]. Other studies report a similar detection rate and confirmed the superiority of FDG-PET in identifying additional metastatic lesions [53,54]. Common serum tumour markers currently have no diagnostic, prognostic, or predictive value for CUP due to their unspecific expression [55]. Table 1 summarizes the imaging techniques used in the diagnosis of BM.

## 4. Pathogenesis of CUP 

The difficulty in identifying the primary tumour might imply that the latter is undetectable either at diagnostic testing or at autopsy, or that CUP constitutes a distinct entity, which challenges classical models of metastasis pathogenesis. Most of the biological steps that are believed to contribute to the pathogenesis of CUP are hallmarks of cancer in general and shared with other neoplastic diseases: chromosomal alterations, self-sufficiency in growth signals, resistance to growth inhibitory signals, reprogramming of energy metabolism, evasion of apoptosis, unlimited replicative potential, bright angiogenesis, tissue invasion, metastasis, and evasion of immune attack [55]. 

A high degree of chromosome instability (CIN) seems to be related to the characteristics of CUP, including aggressive evolution, early metastatic spread, and resistance to chemotherapies [56]. Moreover, CIN might explain the pathogenesis of CUP: a current hypothesis is that the high degree of CIN in CUP metastatic sites makes CUP metastasis resistant to immune surveillance [57], whereas the corresponding less chromosomally instable primary tumour may have regressed. Therefore, the lack (or regression) of a primary tumour in CUP could be interpreted in some cases as an immune-mediated event [58]. An alternative or complementary hypothesis considers CUP metastases in a certain organ as an immunologically edited version of a tumour arising from the same organ. Thus, CUP metastases may constitute a phenotypically modified primary tumour lacking tissue identification and resulting from epitope immunoediting. Familial clustering and association of metastatic location with the affected organ system in family members seem to support this hypothesis [59].

## 5. The Role of Immunohistochemistry 

According to EANO guidelines [51], a tissue diagnosis is mandatory in patients with suspected BM on MRI and unknown primary tumour after a systemic workup before any treatment is undertaken. The histological, ultrastructural, and IHC features of BM usually reflect the characteristics of the primary tumour: therefore, these analyses can be helpful in cases of CUP in the attempt of identifying the primary lesion (Table 2). However, in 11% of patients with BM, the primary tumour remains unknown despite extensive IHC and molecular techniques [35]. In studies on BM-CUP, adenocarcinoma was the most common histological type (235/370 lesions, 63.5%), followed by squamous cell carcinoma (SCC) (37/370, 10%) [4,5,6,7,10,14,17,18,23,27,28,44,60,61] (Table 3). Giordana et al. [5] analysed 99 patients with BM as the first manifestation of cancer using antibodies to carcinoembryonic antigen (CEA) for gastrointestinal or lung cancer, carbohydrate antigen (CA)19.9 for gastrointestinal cancer, cytokeratin (CK) 20 for colon cancer, CA125 for ovarian cancer, BCA-225 for breast cancer, PSA for prostatic cancer, and HMB-45 for melanoma. Among BM-CUP, 20/26 were intensely positive for CEA, 2 for CEA and CA125, and 11 for CEA and CA19.9. None were positive for either CEA, CA19.9, CA125, PSA, or HMB-45. Overall, the immunophenotypes of BM-CUP resembled BM from non-small lung cancer (NLSC) or from colon cancer, but no definite conclusion about the origin was drawn. In the study by Drliceck et al. [39], a combination of antibodies against different CK for epithelial markers (AE1/AE3 (a keratin cocktail that detects CK1-8, CK10, CK14–16, and CK19); CK7, CK10/13, CK18, and CK20), vimentin (epithelial cancers), protein S100 (melanoma), TTF-1 (lung), CA 15-3 (breast), CA19.9, CA125, and PSA was used to identify the primary origin of BM. The primary tumour was identified in 37 out of 54 (68.5%) BM, 29/40 (72.5%) in BM associated with clinically known primary, and 8/14 (57.1%) in BM-CUP. The authors established a first-line panel to analyse BM-CUP, consisting of CK AE1/AE3, CK7, CK18, CK20, vimentin, protein S100, TTF-1, and CA 15-3. In case of positivity for CA 15-3, markers, like CA125 and CA19.9 were suggested to confirm the breast as primary tumour; if the first-line investigation is inconclusive, surfactant (lung) and PSA (prostate) should be added. 

## 6. Gene Expression Profiling: A New Frontier 

In recent years, gene expression profiling (GEP) has become an additional tool for diagnostic, prognostic and predictive purposes. Second-generation microRNA-based assays, GEP-based microarray tests, or quantitative-PCR (qRT-PCR) low-density arrays have reached a sensitivity of 77–94% in identifying CUP [48,55]. Results from a blinded series of high-grade metastatic cases demonstrated the superior accuracy of a 92-gene assay versus standard-of-care IHC, supporting the diagnostic utility of a molecular investigation in difficult-to-diagnose metastatic cancer [62]. Nevertheless, scepticism persists regarding the use of GEP in CUP, as frequent mutations in CUP include TP53, RAS, CDKN2A, MYC, ARID1A, PIK3CA, and BRAF, which are not tissue specific. Moreover, significant discrepancy has emerged between the suspected primary tumour based on molecular profiling and autoptic confirmation. Finally, tumour cells may undergo extensive immunoediting (see the pathogenesis section), which raises doubts over the accuracy of a diagnosis based on GEP positivity [55,58]. Applications of these techniques to BM-CUP have been limited. Mueller et al. described a microRNA-based test (48 microRNAs) that classified 84% (75 of 89) of BM of known primary. When applied to central nervous system (CNS) metastasis from CUP (CNSM-CUP), the test prediction was in agreement with the diagnosis, either clinically or pathologically confirmed, in 80% of cases [35]. An updated version of the assay (64 microRNAs) reached an overall sensitivity of 85%, measured blindly on a validation set of 509 independent samples. Moreover, a clinical validation study on 52 CNSM-CUP patients showed an 88% concordance [34]. A method based on DNA methylation profiles has recently been used to distinguish gliomas from BM, and has been tested in the identification of the origin of BM with known primary, correctly classifying 95% of the samples. Data on BM-CUP are not available [63]. Liquid biopsies collect and analyse circulating tumour cells (CTCs) and cell-free tumour (ctDNA) in body fluids (such as cerebrospinal fluid (CSF) and plasma); these can be a surrogate of tumour tissue in the management of both primary and secondary brain tumours [64]. For example, CSF ctDNA analysis has been successfully used in a case of multiple leptomeningeal and spinal cord metastases from CUP [65] (this case is discussed below). 

Indeed, a promising application of GEP in BM-CUP (and CUP in general) relies on the possibility of identifying targetable molecular markers, rather than identifying a suspected primary site. This is motivated not only by a recent hypothesis in the pathogenesis of CUP but more importantly by recent advances in target therapies. 

## 7. Management 

### 7.1. Outcome

CUP patients can be divided into two prognostic subgroups according to clinicopathological characteristics: a minority of patients (15–20%) have a favourable prognosis and achieve a median survival of 10–16 months and long-term disease control in 30–60% of cases; conversely, the majority of patients have an unfavourable prognosis with a survival of 3–6 months despite a variety of chemotherapeutic combinations. Unfavourable prognostic features include adenocarcinomas and undifferentiated tumours, male gender, age ≥65 years, poor performance status, multiple comorbidities, metastases involving multiple organs, nonpapillary malignant ascites, peritoneal metastases, multiple cerebral metastases, and multiple lung/pleural or bone lesions [3]. In patients with a favourable prognosis, the clinical behaviour, response to treatment, and outcome are similar to those of metastatic tumours from a known primary site [50].

The median survival for BM-CUP ranges between 3 and 12 months (Table 4), although longer survival is reported (Table 5). The prognosis of BM-CUP in comparison with BM from known primaries is debated. Although the majority of studies did not report significant differences in survival between patients with BM of known or unknown origin [8,14,17,19,21,22,25,30,66,67], older studies reported a better overall outcome in BM-CUP ascribed to the lack of a significant extracranial tumour burden [10,18]. In a recent study by Füreder et al. [24], patients with BM-CUP showed a better survival when the primary was identified (after a latency of > 3 months). This was attributable to late systemic progression and the feasibility of tumour-specific chemotherapy. In general, the presence of extracranial metastasis, age, location and number of BM, and performance status emerge as prognostic factors in most studies (Table 4). 

### 7.2. Standard Treatments 

Current treatments of BM include surgery (for tissue diagnosis, symptomatic relief, and in selected cases prolonging survival), stereotactic radiosurgery (SRS), radiotherapy (RT), and/or systemic medical therapies [51]. The management of CUP is tailored according to clinico-pathological subgroups with distinct prognosis [50]. Data from clinical trials in CUP are scarce and in daily clinical practice, when a primary tumour is not histologically characterized but highly suspected on clinical grounds and IHC, it is accepted to treat the patient accordingly to the putative primary [58]. Updated indications for the treatment of CUP are available in the NCCN guidelines [2]. Data on the treatment of BM-CUP come from retrospective studies that include a small number of patients over a long time, thus it is difficult to compare. Systemic chemotherapy with conventional agents in BM-CUP does not achieve a long-term benefit and the overall survival is estimated to reach 12.3% after 2 years, 6.6% after 3, and 0% after 5 [68]. Most authors recommend local treatments (surgery or stereotactic radiosurgery (SRS)), although no conclusions on survival can be drawn (Table 4). Importantly, in a recent series, most patients died due to extracranial progression, and only a few older studies report a majority of deaths due to intracranial progression [16,23]. Relevant aspects to consider when programming local treatments are the number, volume, and location of BM as well as the performance status of the patient. SRS is reported as an effective and safe therapy in BM-CUP, with the advantage of being performed even in elderly patients or in those who are not amenable to surgery [8,17,25,27,30,31,36,37,40,69]. Some authors advocate total resection in selected patients, reporting a significant difference in prognosis between complete and incomplete resection [14,18,42,43,44,45,46,61].

According to less recent studies, whole-brain radiotherapy (WBRT) improves survival in BM-CUP [10,14,16,42,43]. Yuile et al. [41] reported a significant survival benefit with time/dose fractionation (TDF) > 70 in patients with BM-CUP, regardless of age. Rades et al. [32] compared short-course radiotherapy (RT) (20 Gy in 5 fractions) with extended regimens, and did not find any difference in local control and survival. In a more recent study, surgery plus WBRT with boost was associated with a better outcome on univariate analysis, although on multivariate analysis, only the performance score and extra-cerebral lesions retained significance [38]; in a study conducted on patients over 65 years old and treated with WBRT for BM-CUP, a Karnofsky performance score (KPS) ≤ 60 and presence of non-cerebral metastases were associated with unfavourable survival [70]. 

### 7.3. Innovative Treatments: Case Reports

Evidence from clinical trials in CUP is patchy and scarce, due to difficulties in patient recruitment and heterogeneity of cohorts. The problem of a lack of studies is even more evident in the subgroup of BM-CUP due to the rarity of cases, unfavourable prognosis, and frequent exclusion from clinical trials. Therefore, most information on innovative treatments comes from case reports [65,71,72,73,74]. Even if anecdotal, these reports offer good insight on feasible therapeutic strategies and potentially successful treatments in this rare and fatal disease (Table 5). Innovative local approaches include the treatment of a melanoma BM of unknown origin with ECHO-7 Oncolytic Virus Rigvir following surgical resection with a negative 3-year-follow-up [72] and a case of multiple symptomatic BM-CUP, who underwent surgical resection and cesium-131 (Cs-131) brachytherapy to one lesion and SRS to the other two lesions, with a negative follow-up after 6 years [73]. 

**Table 5:** Case reports of brain metastasis from cancer of unknown primary (BM-CUP) reporting innovative therapies or prolonged survival.

Ref	Age	Presenting Symptoms	Location	Histopathology	Other Findings	Treatment	Primary Found during Follow-Up	Survival
Yamasaki et al., 2018 [71]	67	Right lower extremity paresis, dysarthria, memory loss.	Bilateral multiple cerebral lesions	Adenocarcinoma CK7 and TTF-1 +,	147 pack-year smoking history. CEA level029.6ng/mL	Oral erlotinib (150 mg/day).	No	>8 months. Significant improvement of brain and other metastases
Huang et al., 2018 [65]	35	Headache and lower back pain	leptomeningeal metastases + spinal cord	Adenocarcinoma CK7, CDX2, and PAX-8 + TTF-1, CK20, ER/PR – No ALK rearrangements, EGFR and BRAF mutations, microsatellite stable. FISH demonstrating HER2 positivity on dual probe (HER2/CEP17 ratio 5.08).	CSF cytology = ctDNA analysis found amplifications of HER2 and MPL, mutations in the PIK3CA, CDKN2A and TP53 genes	Ado-trastuzumab emtansine + intrathecal trastuzumab and oral lapatinib	No	Clinical improvement, reduction of tumour markers and negative follow-up CSF cytology. >2 years
Proboka et al., 2018 [72]	60	Dizziness after movements and increased fatigue	Brainstem	Metastatic melanoma. weakly positive for HMB-45 and Melan A, strongly positive for MART-1, S-100, and vimentin, and that the Ki-67 index was 35%. BRAF gene mutations in codons V600E, V600K, and V600D were not detected.	NS	Surgery + ECHO-7 Oncolytic Virus Rigvir	No	Follow-up imaging was stable > 3 years
Mahase et al., 2017 [73]	84	Sudden-onset dysgraphia, right limbs weakness, confusion	Multiple bilateral lesions	Adenocarcinoma CK7, TTF-1 ER + CK20, PR, HER2-neu, CDX-2, WT-1, EGFR -	NS	SRS+ Craniotomy + implantation of Cs-131 in the surgical cavity	No	Alive at 6 years follow-up
Kuwata et al., 2011 [74]	69	Dizziness	Cerebellar + multiple smaller brain tumours	Adenocarcinoma, Napsin A and TTF-1 + EGFR exon 19 deletion	Serum CEA= 129.9 ng/mL; SLX = 150 U/mL. Other tumour markers normal range.	Surgical resection of cerebellar lesion + GKRS on other. Gefitinib (250 mg/day).	No	At 46 days follow-up shrinkage of LN and reduction of markers. Survival NS

CPA = cerebello-pontine angle; IAC = internal auditory canals; ECGDS = esophagogastroduodenoscopy; CEA = carcinoembryonic antigen; T-PSA = Total prostate-specific antigen; SCCAg = squamous cell carcinoma antigen; TTF1 = thyroid transcription factor-1; EGFR = epidermal growth factor receptor; CK7 = cytokeratin 7; ER= oestrogen receptor; CK20 = cytokeratin 20; PR = progesterone receptor; HER2-neu = human epidermal growth factor receptor 2; CDX-2 = caudal type homeobox two; WT-1 = Wilm’s tumour 1; GFAP = glial fibrillary acidic protein; SRS = Somatostatin receptor scintigraphy.

In the era of precision medicine, as druggable molecular targets exist across different tumour types, “primary-agnostic” strategies in CUP are feasible. Indeed, clinically relevant mutations can be identified in up to 85% of CUP patients and 13–64% of these are potentially targetable [48]. Yamasaki et al. [71] reported a case of multiple BM-CUP with histopathological diagnosis of adenocarcinoma, with the presumed primary site (not confirmed) in the lung. EGFR exon 19 deletion and programmed death-ligand 1 (PD-L1) expression were detected. Following treatment with oral erlotinib, an EGFR tyrosine kinase inhibitor (TKI), at 150 mg/daily, the symptoms improved and the lesions in the brain, lymphnodes, and duodenum shrank. At the time of publication, the patient was alive with no complaints and no disease progression and was on continuous erlotinib for 8 months. Reduction of lymph nodes metastases and serum tumour markers have also been reported in a BM-CUP (positive for Napsin A, TTF-1, and EGFR exon 19 deletion) treated with the EGFR-TKI gefitinib [74]. Huang et al. performed CSF ctDNA analysis in a case of multiple leptomeningeal and spinal cord metastases from CUP [65]. Next-generation sequencing (NGS) revealed amplification of HER2 and MPL, as well as mutations in the PIK3CA, CDKN2A, and TP53 genes. Considering these results, suggesting a breast primary tumour, ado-trastuzumab emtansine (TDM1) was added to the combination regimen of intrathecal trastuzumab and oral lapatinib. A dramatic clinical improvement, with a reduction of tumour markers and negative CSF cytology (in three subsequent spinal taps), was reported. The patient was alive at the 2-year follow-up, with the primary tumour still undiscovered. However, apart from these isolated case reports, there is currently no further evidence of improved outcome with the use of tumour-specific therapy guided by molecular testing in CUP patients [2].

### 7.4. Innovative Treatments: Ongoing Clinical Trials 

BM-CUP has usually been excluded in trials on targeted therapies conducted in CUP (e.g., NCT00894569, NCT04131621) or has been accepted with strict inclusion criteria, such as “non-active BM”, “non-symptomatic BM”, or “only if the lesions had been previously treated with surgical resection or radiation therapy and were steroid independent with minimal residual symptoms”, thus precluding any generalization [75,76] (Table 6). GEFCAPI04 is a phase III trial in Europe, which compares treatment strategies based on GEP results to conventional cytotoxic therapies in patients with CUP, including patients with stable brain metastasis (NCT01540058). The CUPISCO trial (NCT03498521) is a phase II randomized study that evaluates the efficacy and safety of targeted therapies vs. standard chemotherapy in CUP, including BM—but not leptomeningeal metastasis—from CUP. The role of immune checkpoint inhibitors in CUP is currently being assessed in prospective trials; however, the eligibility of BM-CUP is limited to those previously treated and stable (NCT02721732, NCT03391973, NCT02834013, NCT03752333, NCT03396471). NCT04273061 is a phase II study (not yet recruiting) whose aim is to investigate the effects of PDL1 inhibitor atezolizumab in tumours in which GEP suggests sensitivity to treatment, including CUP and BM if asymptomatic and stable (Table 5). Other trials, such as the TAPUR (NCT02693535), MPACT (NCT01827384), IMPACT II (NCT02152254), and the Match Screening (NCT02465060), are evaluating the efficacy of a biomarker-based approach in solid tumours in general, including patients with stable BM from CUP (Table 6).

## 8. Conclusions

BM-CUP is a clinical challenge and there is still a lack of specific diagnostic tools and therapies. The advancement of knowledge in molecular biology will lead to the identification of an increasing number of pathways to be successfully targeted with molecular agents. Hopefully, this could improve the prognosis of these patients and allow the normal brain to be spared from damage.

## Figures and Tables

**Table 1:** Imaging techniques for the diagnosis of brain metastases (BM).

**Contrast-Enhanced Computed Tomography (CT) Scan**	Can be performed in emergency, no contraindications (except pregnancy and allergy to contrast medium). Allows visualization of acute bleeding. Less sensitive than MRI (especially for lesions in the posterior fossa, multiple punctate metastases and for leptomeningeal metastases)
**Contrast-enhanced magnetic resonance (MRI)**	Higher resolution then CT scan; needs patient’s collaboration (not suitable in case of psychomotor agitation, claustrophobia). Contraindicated in patients with medical devices that are not compatible (some types of pacemaker, metallic implants, etc.) and if there are contraindications to the contrast medium (allergy, risk of nephrogenic systemic fibrosis, etc.).
**Diffusion-weighted (DW)-MRI**	Useful in differential diagnosis with abscesses: diffusion usually restricted in abscesses and unrestricted in BM. Exception: mucinous BM can show restricted diffusion
**Gradient echo/other susceptibility-weighted images (SWI)**	Useful for the identification of hemosiderin and other blood breakdown products. May improve detection of haemorrhagic BM.
**Perfusion MRI - cerebral blood volumes (CBVs)**	Peri-tumoral CBV lower in BM than in malignant gliomas, while higher in BM than in abscesses
**MRI spectroscopy (MRS)**	Lower choline/creatinine ratios in BM compared to high grade gliomas.
**18F-fluordesoxyglucose (FDG)-Positron emission tomography (PET)**	Lower sensitivity and specificity than MRI in the detection of BMs. Does not provide enough information for differential diagnosis. Whole body FDG-PET useful to identify the primary tumour or other metastasis (see main text).

**Table 2:** Most used immunohistochemical markers for brain metastasis.

Squamous Cell K.	CK5/6, CK7, EMA, GFAP
Small cells K of the lung	CD56, CK7, TTF1, EMA
Lung Adeno-K	CK7, TTF1, Napsin A, EMA, CAM 5.2, CEA, RCC (Variable)
Breast Adeno-K	CK5/6 (Variable), CAM 5.2, CK7, GCDFP15, EMA, S100 (variable), CEA, CA 15-3
Endometrial K	CAM 5.2, CK7, CA 125, ER (variable), CA125, CEA
Colorectal Adeno-K	CAM 5.2, CK20, CDX2, EMA, CEA, CA 19.9
Stomach Adeno-K	CAM 5.2, CK7, CK20, CDX2, EMA, CEA, CA 19.9
Prostate Adeno-K	PSA, EMA
Urothelial K	CK5/6, CK7, CK20, EMA
Renal cell K	RCC, EMA, PAX8, Vimentin
Melanoma	Vimentin, Melan A, S100, HMB 45

K = carcinoma; Adeno-K= adenocarcinoma; CK = cytokeratin; EMA = epithelial membrane antigen; TTF1 = Thyroid transcription factor 1; PSA = prostate-specific antigen; RCC = renal cell carcinoma protein; CEA = Carcinoembryonic antigen; CA = carbohydrate antigen.

**Table 3:** Histological diagnosis and immunohistochemical (IHC) markers in the diagnosis of brain metastases from cancer of unknown primary (BM-CUP).

Studies	Histological Diagnosis	Immuno-Histochemical Markers Used	Confirmed Primaries at Follow-Up
Matsunaga et al., 2019 [27]	42 Adeno-K, 4 squamous-cells, 2 neuroendocrine	NS	NS
Mavrakis et al., 2005 [6]	7 Adeno-K, 2 poorly differentiated malignant epithelial tumours	NS	2/9 (22%)
Drlicek et al., 2004 [39]	Lung 5, colorectum 1, breast 1, kidney 1	CK (AE1/AE3, 7, 10/13, 18, 20), vimentin, protein S100, TTF-1, and CA 15-3, 19.9, 125, PSA	0
Bartelt and Lutterbach, 2003 [14]	15 Lung Adeno-K,4 Squamous-cells, 4 Large-cells, 4 Small-cells, 20 Other	NS	NS
Klee et al., 2002 [60]	14 Adeno-K (12 CK 7 +, none CK 20), 1 carcinoma (no CK 7, CK 20 and CK), 1 melanoma	CK7, CK20, PSA, HCG, CA125 and “antibodies indicating breast or pulmonary primary”	7/14 (50%)
Rudà et al., 2001 [61]	4 Lung Adeno-K, 1 Squamous-cells, 3 Colon Adeno-K, 1 Pancreatic Adeno-K, 18 no diagnosis	NS	27/33 (81%)
Maesawa et al., 2000 [17]	10 Adeno-K, 2 Squamous-cells, 1 Clear-cells, 2 Undifferentiated	NS	4/15 (26.7%)
Nguyen et al., 1998 [18]	31 Adeno-K, 2 small-cells,1 squamous-cells, 4 other, 1 missing	NS	12/39 (31%)
Salvati et al., 1995 [10]	65 Adeno-K, 10 Squamous-cells, 10 Melanoma, 7 Undifferentiated, 7 other small-cells	NS	64/100 (64%)
Debevec, 1990 [44]	Anaplastic K and adeno-K were most frequent	NS	47/75 (63%)
Merchut, 1989 [7]	8 Adeno-K + 1 squamous-cells	NS	47/56 (84%)
Chee and Byrnes, 1988 [4]	5 Adeno-K, 3 anaplastic, 4 squamous-cells, 1 sarcoma, 1 transitional-cells	NS	35/51 (68%)
Eapen et al., 1988 [23]	9 No diagnosis, 19 Adeno-K, 7 Squamous K, 5 Anaplastic K, 1 large-cells, 1 small-cells, 1 transitional-cells	NS	11/43 (25%)
Zimm et al., 1981 [28]	14 Adeno-K, 2 squamous	NS	10/16 (37%)

NS = not specified, IHC = immunohistochemistry, K = carcinoma, Adeno-K = adenocarcinoma.

**Table 4:** Treatment and prognosis of brain metastasis from cancer of unknown primary (BM-CUP) in retrospective studies.

Studies	N of BM- CUP	Treatment of BM	Survival	Local Control	Causes of Death	Determinants of Survival
Gough et al., 2020 [66]	55	Surgery	Median survival = 6 m; survival at 1 y = 29.1%; at 5 y = 0.	Median time to recurrence = 6 m	NS	Decreased survival associated with other metastases, older age and ECOG score. No significant difference between CUP Vs known primary tumour.
Matsunaga et al., 2019 [27]	152	GKRS	Median survival = 6 m; survival at 6 m = 79.3%, at 12 m = 14.9%	Response at 6m: complete = 4.4%, partial = 74.2%, stable = 13.3%, progressive disease = 8.1%	Systemic progression = 67% brain progression = 33%;	Decreased survival associated with higher age, lower KPS score, extracranial MTS, multiple BM
Dziggel et al., 2018 [31]	8	SRS	Survival at 6 m = 63%; at 12 m = 63%. Median survival time was not reached during the follow up.	Local control 100% at 12 m; freedom from new cerebral lesions at 6 m = 86%, 10 m = 64%	NS	Improved survival associated with male gender, single BM
Rades et al., 2018 [38]	140	WBRT	Survival at 6 m = 33%; at 12 m = 18%.	Local control: 6 m = 36% 12 m = 24%.	NS	Improved survival associated with ECOG-score, extra-cerebral lesions
Han et al., 2016 [25]	10	GKRS	Median survival = 35.3 m	Median times to new lesion detection = 4.6 months	Systemic progression = 79%, brain progression = 16%; unrelated cause = 5%	NS. No significant difference between CUP Vs known primary tumour
Niranjan et al., 2010 [8]	29	SRS	Median survival = 12 m	Local control after 18.6 m = 88.5%. Progression free survival at 6 m = 96.4%, at 12 m = 81.9%	Systemic progression = 90%, brain progression = 10%	Decreased survival associated with location in the brainstem
Yamamoto et al., 2009 [36]	32	GKRS	Median surviva l = 6.5–7 m	NS	NS	Improved survival associated with n of lesions, tumour volumes, non-symptomatic, well-controlled primary, no extracranial MTS, KPS > 80%, >2 procedures
Rades et al., 2007 [32]	101	WBRT=Long course (10 × 3 Gy) Vs short-course WBRT (5 × 4 Gy)	Median survival in RPA-I = 7.1 m, in RPA-II = 4.2 m, in RPA-III = 2.3 m	Local control at 6 m: short WBRT = 80%, long WBRT = 50%	NS	Improved survival associated with KPS > 70, no extracranial metastases, RPA-class = 1
D’Ambrosio and Agazzi, 2007 [22]; Agazzi et al., 2004 [19]	35	WBRT + Surgery/SRS	Median survival (after diagnosis) = 3.2 m	NS	Systemic progression in most (NS)	No significant difference between CUP Vs known primary. In CUP, no difference if identification of the primary
Bartelt and Lutterbach, 2003 [14]	47	WBRT	Survival at 3m = 68%, at 6 m = 39%, at 12 m = 26%, at 24 m = 5%	NS	NS	Improved survival associated with KPS > 70 and resection. No significant difference between CUP Vs known primary tumour.
Petrovich et al., 2002 [40]	14	GKRS	Median survival = 6 m	NS	Systemic progression = 70%, brain progression = 23%, unknown = 7%.	Improved survival associated with KPS > 70, less active systemic disease, total intracranial tumour volume (<3 cm)
Yuile and Tran, 2002 [41]	25	WBRT	Median survival = 4 m	NS	NS	Improved survival associated with Radiation dose >40 Gy, degree of surgery, primary site (lung)
Rudà et al., 2001 [61]	33	WBRT + surgery	Median survival = 10 m; Survival at 6 m = 76%, at 12 m = 42%, at 24 m = 15%	NS	NS	Improved survival associated with single BM
Hall et al., 2000 [42]	34	WBRT	Survival (after diagnosis): at 2 y = 12.3%, at 3 y = 6.6%, at 5 y = 0%	NS	Systemic progression in most (NS)	Improved survival associated with younger age, single of BM, surgical resection, WBRT, CT
Maesawa et al., 2000 [17]	15	SRS	Median survival = 15 m survival at 2 y = 53.3%, at 3 y = 20%	Crude local tumour control rate = 92.6%. Tumour control rate at 4 y = 91.3%	Systemic progression = 53%, Brain progression = 20%, unknown = 27%	Improved survival associated with BM location (other than brainstem), extracranial disease. Detection of the primary site did not affect survival
Lagerwaard et al., 1999 [16]	102	Surgery and/or RT	Median survival (after diagnosis) = 5.5 m; Survival at 6 m = 48%, at 1 y = 22%, at 2 y = 10%	NS	Brain progression = 57%.	Improved survival associated with surgery+RT, ECOG, response to steroid treatment, systemic activity, serum LDH, unknown primary, age, number of BM
Nguyen et al., 1998 [18]	27	WBRT + surgery	Median survival = 13.4 m Survival at 12 m = 56%, at 18 m = 38%, at 5 y = 15%, at 8 y = 12%	5 y = 72%	Systemic progression in most (NS)	Improved survival associated with gross total resection and RT
Nussbaum et al., 1996 [43]	33	WBRT + surgery/CT	Median survival (after diagnosis) = 7 m	NS	NS	Improved survival associated with surgery, RT, CT and younger age.
Salvati et al., 1995 [10]	100	Surgery	Survival at 6 m = 43%, at 1 y = 19%	Intracranial progression/relapse = 60%	Perioperative mortality = 6%; brain progression = 38%; systemic progression = 66%	Improved survival associated with unknown primary, RT, number of MTS in different organs <2
Debevec, 1990 [44]	75	WBRT	Median survival = 9.5 m; survival at 1 y = 41%.	NS	Brain progression = 60%	NS
Merchut, 1989 [7]	56	WBRT/surgery	Survival at 6 m = 55%, at 1 y = 13%	NS	NS	NS
Eapen et al., 1988 [23]	43	Surgery and/or WBRT	Survival at 6 m = 52%, 12 m = 20%	NS	Brain progression = 68.3%, systemic progression = 31.7%	Improved survival associated with single BM
Chevalier et al., 1985 [21]	67	Surgery and/or WBRT	Survival (after diagnosis) at 6 m = 44%, at 12 m = 16%, at 24 m = 5%	NS	NS	NS. No significant difference between CUP Vs found primary tumour
Yardeni et al., 1984 [45]	26	Surgery	Median survival = 3.5 m, survival at 1 y = 19.2%, 2 y = 11.5%	NS	NS	NS. No correlation between survival and age or extracranial metastasis.
Zimm et al., 1981 [28]	16	Surgery + WBRT/CT ^1^	Median survival (after diagnosis) = 7.2 m	NS	Brain progression = 75%	Improved survival associated with younger age, single BM, ambulatory performance status <1, presenting symptoms (headache, personality change, visual disturbances)
Ebels and van der Meulen, 1978 [46]	19	Surgery	Median survival = 6.8 m	NS	NS	NS

Median survival is intended as after treatment where not specified. GKRS = gamma knife radiosurgery; SRS = stereotactic radiosurgery; WBRT = whole brain radiotherapy; MRI = magnetic resonance imaging; NS = not specified; n = number; m = months; y = years; MTS = metastases; KPS = Karnofsky Performance Status; RPA=recursive partitioning analysis score; ECOG = eastern cooperative oncology group performance score; CT = chemotherapy; LDH = lactate dehydrogenase; ^1^ = intracarotid artery infusions of BCNU, 70–90 mg/m^2^.

**Table 6:** Clinical trials on targeted therapy in cancer of unknown primary (CUP) that include brain metastasis.

Table	Type of Study	Intervention	CUP Inclusion Criteria	BM Inclusion Criteria	Primary end Point
**NCT01540058**	Phase 3	Experimental: test-guided strategy (primary cancer suspected by “the BioTheranostics Cancer Type ID test” molecular analysis) Vs. Active Comparator: Empiric strategy (Gemcitabine/Cisplatin)	Histopathological confirmed (with IHC): moderately or well-differentiated adenocarcinoma, poorly differentiated adenocarcinoma, undifferentiated carcinoma, squamous-cell carcinoma	Not symptomatic	Progression free survival (death/RECIST criteria)
**NCT03498521**	Phase 2 Randomized	Experimental: Molecularly Guided Therapy ^1^ (based on genomic profile) Vs. Active Comparator: platinum-based chemotherapy (Carboplatin or Cisplatin in combination with Gemcitabine or Paclitaxel)	CUP diagnosed according to criteria defined in the 2015 ESMO Guidelines	Previously treated BM without residual disease or leptomeningeal disease	Progression free survival (death/RECIST criteria)
**NCT03396471**	Phase 2 Single-arm	Pembrolizumab + External Beam Radiation Therapy	CUP after complete negative diagnostic workup	Previously treated BM, stable >4 weeks, no new or enlarging BM, no steroids for > 7 days. No carcinomatous meningitis.	Response rate (irRECIST and RECIST criteria)
**NCT02721732**	Phase 2 Single-arm	Pembrolizumab	Advanced (unresectable and/or metastatic) solid tumour (including CUP) that has progressed following standard therapies (if available)	Previously treated BM, stable >4 weeks, no new or enlarging BM, no steroids for > 7 days. No carcinomatous meningitis.	Non-progression rate (irRECIST and RECIST criteria)
**NCT04273061**	Phase 2 Single-arm	Atezolizumab	Incurable solid tumour (including CUP) with whole genome and transcriptome analysis	Asymptomatic, no SRS < 7 days, WBRT<14 days, resection < 28 days, no ongoing corticosteroids. Stable anticonvulsant therapy permitted.	Overall response rate (RECIST criteria)
**NCT03752333**	Phase 2 Single-arm	Pembrolizumab	CUP after complete negative diagnostic workup. Both first line and previously treated patients.	Previously treated BM, stable >4 weeks, no new or enlarging BM, no steroids for > 7 days. No carcinomatous meningitis.	Overall response rates by (irRECIST and RECIST criteria)
**NCT02834013**	Phase 2 Non-Randomized Parallel Assignment	Arm I (nivolumab + ipilimumab); Arm II (nivolumab)	Histologically and/or biochemically confirmed rare cancer (including CUP after complete negative diagnostic workup)	Treated >= 28 days, off steroids > 7 days	Overall response rate (RECIST criteria)
**NCT03391973**	Phase 2 Single-Arm	Pembrolizumab	CUP after complete negative diagnostic workup	Previously treated BM, stable >4 weeks, no new or enlarging BM, no steroids for > 7 days. No carcinomatous meningitis.	Objective response rate (RECIST criteria)
**NCT04273061**	Phase 2 Parallel Assignment	Atezolizumab	Incurable solid tumour (including CUP) with whole genome and transcriptome analysis	Asymptomatic, no SRS < 7 days, WBRT<14 days, resection < 28 days, no ongoing corticosteroids. Stable anticonvulsant therapy permitted	Objective response rate (RECIST criteria)

ESMO = European Society for Medical Oncology, RECIST = Response evaluation criteria in solid tumours; irRECIST= immune-related Response Evaluation Criteria in Solid Tumors; ^1^ Molecularly Guided Therapies = Alectinib, Vismodegib, Ipatasertib, Olaparib, Erlotinib, Bevacizumab, Vemurafenib, Cobimetinib, Trastuzumab, Pertuzumab, Atezolizumab, Carboplatin, Paclitaxel, Cisplatin, Gemcitabine, Entrectinib.
